# Linking Canadian Administrative Data: Income Trajectories, Residential and School Mobility, and Grade 3 Academic Achievement.

**DOI:** 10.23889/ijpds.v7i3.1812

**Published:** 2022-08-25

**Authors:** Janelle Boram Lee, Elizabeth Wall-Wieler, Leslie L Roos

**Affiliations:** 1 University of Calgary; 2 Manitoba Centre for Health Policy, University of Manitoba

## Objectives

The objective is to examine the association between trajectories of childhood residential and school mobility and academic achievement (literacy, numeracy) in Grade 3 using linked whole-population administrative data in Manitoba, Canada. Secondarily, we assessed childhood residential/school mobility based on neighbourhood income levels (moving in/out of low- or mid-/high-income neighbourhoods).

## Approach

This retrospective cohort study used linkable, de-identified administrative data (health, education, national census, provincial survey) from the provincial Population Research Data Repository housed at the Manitoba Centre for Health Policy (MCHP). Among kindergarteners from 2005 to 2014 (n = 83,894), those not having continuous residency in Manitoba, valid education assessments, and relevant family-level covariates were excluded. We followed this eligible cohort from kindergarten to Grade 3 based on various neighbourhood income trajectories of residential and school mobility. To assess Grade 3 literacy and numeracy scores based on trajectories, log-binomial regression models were conducted using SAS® version 9.4.

## Results

The total cohort included 36,754 children; at the end of kindergarten, 14.2% resided in low-income neighbourhoods, and 84.8% lived in mid-/high-income neighbourhoods. Moving between two low-income neighborhoods between kindergarten to Grade 3 was associated with an increased risk of poor Grade 3 numeracy and literacy scores (numeracy aRR=1.39 [1.16,1.67]; literacy aRR=1.31 [1.08,1.59]). When moving between neighborhood income levels, the association was stronger for children moving into low-income neighbourhoods (e.g., mid-/high-income to low-income: numeracy aRR=1.41 [1.19,1.67]) than children moving into mid/high-income neighbourhoods (e.g., low-income to mid-/high-income: numeracy aRR=1.31 [1.08,1.59]). Changing schools between kindergarten and Grade 3 was also associated with poorer numeracy and literacy scores in Grade 3 (numeracy aRR=1.31 [1.22,1.40]; literacy aRR=1.34 [1.24,1.44]); however, the strength varied based on residential mobility patterns.

## Conclusion

Moving homes/schools can differentially impact children’s educational attainment depending upon the income level of residing neighborhood(s). Stakeholders should recognize different levels of risks related to mobility and provide support accordingly to reduce the adverse impact. Support systems should be tailored to not only children but also families and neighbourhoods.

